# Rating of perceived exertion as an indirect indicator of velocity loss during power back squat exercise with moderate loads

**DOI:** 10.3389/fspor.2026.1836628

**Published:** 2026-09-04

**Authors:** Hanye Zhao, Takanori Kurokawa, Masayoshi Tajima, Zijian Liu, Junichi Okada

**Affiliations:** 1 College of Sport and Health Science, Ritsumeikan University, Kusatsu, Shiga, Japan; 2 Institute for Sport Sciences, Waseda University, Tokorozawa, Saitama, Japan; 3 Graduate School of Sport Sciences, Waseda University, Tokorozawa, Saitama, Japan; 4 Rehabilitation Medicine Center, The Second Affiliated Hospital of Wenzhou Medical University, Wenzhou, Zhejiang, China; 5 Faculty of Sport Sciences, Waseda University, Tokorozawa, Saitama, Japan

**Keywords:** muscle fatigue, perceived exertion, power training, surface electromyography, velocity loss

## Abstract

**Introduction:**

It's important to maintain high propulsive velocity and to avoid severe velocity loss during power-aimed resistance exercises. However, it is hard to measure velocity loss in a timely manner without special devices. This study examined the validity of using ratings of perceived exertion (RPE) as velocity loss marker in power back squat (BS) with different repetition settings.

**Methods:**

Seventeen trained males performed three BS tasks with low, medium, and high repetitions at 65% of their one-repetition maximum. RPE, surface electromyography, and concentric velocity were assessed across all conditions. Peak root mean square (RMS) and median frequency (MDF) were calculated from the surface electromyography data.

**Results:**

Significant effects were observed across experimental conditions for average RPE and average velocity loss, (*p* < 0.001), while no significant difference was found in average RMS and MDF. As the lifting tasks progressed, significant effects of repetition were observed in RPE, RMS, and MDF (all *p* < 0.001). When comparing conditions, significant differences were found among the three in RPE and velocity loss (*p* < 0.001), whereas no significant effect of condition was observed in RMS and MDF.

**Discussion:**

RPE was a significant predictor of velocity loss within individuals (*p* = 0.002), and this relationship could be expressed as an RPE of 3.8 for a 10% velocity loss. These findings indicate a device-free reference that can be incorporated into established RPE-based autoregulation of velocity loss threshold during power BS at moderate load.

## Introduction

Power and velocity are two key factors that are closely associated with actual athletic performance in practical scenarios (1). Over the past decades, numerous studies have focused on developing power output and movement velocity through resistance exercises (2, 3). The configuration of power-aimed resistance training has traditionally been associated with several key factors, including load, sets, rest intervals, and the number of repetitions. Almost all related variables have been well researched, and classic prescription guidelines for resistance training have been established (1). For example, in resistance training programs focused on developing muscular power, a standard recommendation is to lift with a moderate load under a moderate repetition scheme and with an explosive movement intent (e.g., 6 repetitions at 45% of one-repetition maximum [1RM]) (4).

Although the external load constrains the actual movement velocity, attempting to move explosively is expected to impose a high neural drive and motor-unit recruitment demand (5). As repetitions are repeated, fatigue-related alterations within the neuromuscular system may reduce the ability to maintain this intended explosive output, thereby resulting in a progressive decline in actual concentric velocity (6, 7). These acute decreases in performance may negatively impact the training stimulus and, subsequently, long-term training adaptations (6). Surface electromyography (sEMG) provides complementary information on changes in muscle activation during repeated contractions (8). Amplitude-based measures such as the root mean square (RMS) are commonly interpreted as reflecting the magnitude of neural drive to the muscle, whereas spectral measures such as the median frequency (MDF) have been used as indirect indices of peripheral changes within the muscle. A concurrent increase in RMS and decrease in MDF has traditionally been regarded as a classic signature of neuromuscular fatigue (9, 10). Therefore, examining the association between velocity loss and sEMG-derived fatigue parameters may help clarify the extent to which impaired explosive performance is accompanied by neuromuscular fatigue-related changes. It should be noted, however, that the association between spectral measures and motor-unit conduction velocity has recently been reported to be weak (11), and that findings obtained under controlled, isometric conditions may not extend to dynamic, high-velocity movements. Nonetheless, RMS and MDF remain the most widely used sEMG indices for characterizing neuromuscular responses during resistance exercise.

Velocity-based training is one of the most commonly used methods for improving explosive performance and many commercial devices are presently available for practical application in training scenarios (12, 13). During velocity-based training, coaches typically monitor the velocity of each repetition throughout the lifting process. When substantial velocity loss is detected or velocity falls outside a predefined velocity zone, the set is terminated and/or the training load is adjusted (14). Linear position transducers are among the most widely used devices for this purpose (13, 15). Regarding measurement precision, most commercially available devices demonstrate acceptable accuracy for both daily condition monitoring and research applications in velocity-based training (15). Although these devices are more affordable than in previous decades, those with acceptable precision remain costly for individual users, such as freelance coaches and trainers. Furthermore, many devices require paid subscriptions for advanced features. These economic barriers limit the adoption of velocity-based training among individual users.

The rating of perceived exertion (RPE) scale is a highly usable tool that is widely used in sports science, exercise physiology, and clinical settings (16). With the increasing global popularity of resistance training, RPE has also been shown to be a valid measure in resistance training–related contexts (17). The original 6–20 Borg scale was developed for aerobic exercise and anchored to heart rate (16). Although both 6–20 and CR-10 scale are validated for rating relative intensity in resistance exercise, heart-rate responses are substantially influenced by repetition number, contraction duration, exercise mode, and inter-set rest intervals (18). Therefore, the CR-10 scale has greater versatility in resistance-exercise scenarios. Given its ease of use as a marker of physiological responses, RPE may also be applied as an indirect marker of velocity loss during velocity-based training.

Accordingly, the purposes of the present study were to (1) examine the criterion-related validity of RPE as an indicator of velocity loss during power BS exercise by modelling the within-participant RPE–velocity-loss relationship, and (2) characterize neuromuscular activation responses (RMS and MDF) during power BS under different repetition settings. Specifically, the present study addresses criterion-related (predictive) validity: whether the RPE reported during a set predicts the concurrent velocity loss within the same individual. We hypothesized that during power BS: (1) RPE scores would exhibit significant relationship with velocity loss, and (2) RPE would increase but would not depend solely on increases in neuromuscular activations.

## Materials and methods

### Experimental designs

This study aimed to examine the validity of using RPE as an indicator of velocity loss and to characterize neuromuscular responses during power BS exercise. The present study included two separate sessions. In the first session, descriptive characteristics, including age, height, body mass, body fat percentage, and 1RM strength, were assessed. After the baseline measurements, all participants were familiarized with the RPE scale. RPE was assessed using the Borg CR-10 scale, which is one of the most widely used RPE models. Subsequently, an anchoring trial was performed for all participants to establish the upper and lower limits of the RPE scale (19, 20). In the second session, participants performed 30% (low repetitions; L), 60% (medium repetitions; M), and 90% (high repetitions; H) of the number of repetitions successfully completed during the anchoring trial (21). The three levels of repetition number were chosen to capture a deliberately spaced gradient of exertion from clearly submaximal to near maximal (22, 23). Three experimental conditions were implemented using a randomized design. To assess velocity loss and neuromuscular responses induced by the lifting tasks, sEMG signals and movement velocity were measured and analyzed.

### Participants

The sample size was estimated using statistical power analysis software (G*Power 3.1; University of Bonn, Germany). An ANOVA model with fixed effects, including main effects and interactions, was selected for the analysis. The effect size was set at 0.40, the alpha level at 0.05, and statistical power at 0.95 (10). The minimum required sample size was 14 participants; accordingly, 17 trained males were recruited. All participants were male college students who regularly engaged in resistance training (training experience: 4.5 ± 2.4 years). None reported a history of neuromuscular injury, nor were they undergoing medical treatment at the time of participation. The descriptive characteristics of the participants (mean ± SD) were as follows: age, 21.4 ± 2.6 years; body mass, 72.6 ± 11.9 kg; body fat percentage, 16.0 ± 3.9%; and BS 1RM, 130.0 ± 33.2 kg. All participants were informed of the purpose of the study, the experimental protocol, measurement procedures, and potential risks. Written informed consent was obtained before the beginning of the first session. The study was conducted in accordance with the Declaration of Helsinki and was approved by the Human Ethics Committee of Waseda University (approval no. 2023-112).

### The first session

Following the ethical briefing, participants’ descriptive characteristics were assessed. Body mass and body fat percentage were measured using a bioelectrical impedance analysis device (InBody 970; InBody Co., Ltd., South Korea). After the assessment of descriptive characteristics, a standardized warm-up protocol was implemented for all participants (7, 24). The warm-up protocol includes: shoulder stretch (10 s/side), chest stretch (10 s/side), thigh stretch (10 s/side), hamstring stretch (10 s/side), calf stretch (10 s/side); dynamic stretch: body weight lunge with trunk twist (10 repetitions/side), self-paced running (5 min), back squat (20 kg and 30 kg for 8 and 6 repetitions, respectively) (7, 24). After the warm-up, the 1RM for power BS was assessed in accordance with established guidelines (1). When performing BS, the participants were instructed to lower until their thighs were parallel with the floor. A pair of laser gates (TCi Timing System, Brower Timing System LLC, USA) was set at the parallel-thigh position to indicate that the participants reached this position during BS.

After the assessment of basic characteristics and 1RM, participants rested for 5 min. During the rest interval, they received detailed instruction on the Borg CR-10 scale, which ranges from ‘nothing at all’ (0) to ‘extremely strong’ (10), with verbal descriptors of perceived exertion (16, 19). Following instruction on the RPE scale, participants were introduced to the prescribed lifting cadence. The eccentric (lowering) phase was performed over 2 s, followed by an explosive concentric (pushing) phase. The cadence was regulated using a smartphone metronome application emitting one beep per second. After a brief rest period, participants completed a practice set using only the barbell, following the prescribed cadence and technique. An anchoring trial was then conducted to establish the subjective exertion range for use in the experimental session. Participants performed a single set of power BS to volitional failure. The low anchor of the RPE scale (score = 0, ‘nothing at all’) was defined as the sensation experienced during a relaxed resting state (25). The high anchor of the RPE scale (score = 10, ‘extremely strong’) was defined as the sensation experienced at the point of volitional failure (26). The relative intensity was set at 65% of each participant's 1RM (27).

### The second session

Before the start of the second session, participants completed the same standardized warm-up routine as in the first session. After the warm-up, participants performed an RPE reporting practice. The Borg CR-10 scale was positioned horizontally in front of the participants to ensure that the numbers and descriptors were clearly visible. The lifting cadence was set in the same manner as in the first session. During the pause between repetitions, participants verbally reported their RPE for the most recent repetition by selecting a number from the Borg CR-10 scale. Participants were instructed to report their RPE based on the anchoring trial conducted in the first session. If perceived exertion exceeded the high anchor (score = 10 on the CR-10 scale), participants were allowed to report values greater than 10 (16, 28).

After the standardized warm-up and practice, the three experimental conditions (L, M, and H) were performed by all participants in a random order. Overall, participants performed 5.4 ± 1.0 repetitions for L, 10.4 ± 2.2 repetitions for M, and 15.8 ± 3.2 repetitions for H conditions. To minimize the influence of the central governor effect, participants were not informed of the required number of repetitions for each condition until the second-to-last repetition (29, 30). Participants were instructed to cease the set after completing one final repetition. RPE scores were reported in the same manner as during the RPE reporting practice. Subsequently, RPE scores were averaged for each condition (26). During the exercise, participants might experience difficulty maintaining the prescribed cadence. In such cases, they were instructed to follow the metronome cues as closely as possible. A 5-min rest interval was provided between conditions.

### Surface electromyography

sEMG signals were recorded from the vastus lateralis and vastus medialis muscles of the dominant leg. For each muscle, two electrodes were placed with an interelectrode distance of 1 cm. Electrode placement followed the standard guidelines for sEMG (31). sEMG signals were recorded using a dedicated wireless system at a sampling frequency of 1,000 Hz (MARQ MQ-8; Kissei-Com Tech, Nagano, Japan). A high-speed camera was positioned in the sagittal plane to record motion video (Grasshopper GRAS-03K2C; FLIR Systems Inc., Canada). Raw sEMG data were collected using proprietary software (Vital-Recorder; Kissei-Com Tech, Nagano, Japan). Only the propulsive (concentric) phases of each repetition were extracted and analyzed. A fourth-order Butterworth band-pass filter (20–450 Hz) was applied to the raw sEMG signals to remove background noise. The concentric (propulsive) phase of each repetition was identified from the vertical displacement of the barbell coordinated. Concentric phase onset was defined as the bar reached the lowest position, and end with the barbell reached the highest position. Root mean square (RMS) amplitude and median frequency (MDF) were calculated as indicators of neuromuscular responses. MDF and peak RMS values were normalized to the first repetition of each condition (32). Subsequently, RMS and MDF values from the vastus lateralis and vastus medialis were averaged into single variables, as these muscles have been reported to exhibit similar activation patterns (33). All RMS and MDF calculations were performed using customized MATLAB scripts (version 2024a; MathWorks, Natick, MA, USA). Neuromuscular fatigue was identified by a significant decrease in MDF accompanied by a significant increase in RMS (10, 34).

### Velocity loss

Barbell velocity during the BS was calculated using a high-speed camera operating at 120 frames per second (fps). The camera was positioned 5 m from the rack to capture high-speed video from the sagittal plane on the participant's left side. A colored marker was attached to the end of the barbell to track its trajectory. A 1-m calibration stick was positioned approximately in the plane of the barbell's vertical path to convert pixel displacement to real distance before the beginning of each condition (35). The propulsive velocity (concentric phase) of the BS was computed frame-by-frame using dedicated motion tracking software (KineAnalyzer, Kissei-Com Tech, Nagano, Japan). The velocity data were segmented into individual repetitions and used for statistical analysis. Instantaneous vertical velocity was computed frame by frame, and the peak instantaneous velocity of each repetition was extracted. Velocity was extracted using custom MATLAB scripts (version 2024a; MathWorks, Natick, MA, USA). To describe the within-set time course, a repetition-wise velocity-loss index was computed by expressing the peak instantaneous velocity of each repetition relative to that fastest repetition (36); because the fastest repetition was not necessarily the first, this index may take non-zero values at the first repetition.

### Statistical analysis

The Shapiro–Wilk test was used to assess the normality of average RPE, average velocity loss, RMS, and MDF for each condition. Average RPE and MDF were normally distributed; therefore, one-way ANOVAs were conducted for these variables, followed by Bonferroni-corrected *post hoc* tests for multiple comparisons. RMS and velocity loss did not fully meet the assumption of normality in some conditions. Accordingly, Friedman's test was employed to assess differences across the three experimental conditions, with Bonferroni correction applied for *post hoc* comparisons.

To investigate changes in velocity loss, RPE, MDF, and RMS during the lifting process, data from specific repetitions were analyzed. The first, median, and final repetitions were extracted for analysis. Two-way ANOVA has been reported to be robust against moderate and even severe departures from normality, particularly in balanced designs with equal sample sizes (37, 38). Therefore, the two-way repeated-measures ANOVA was applied for intra-set data. Sphericity of the variance of the distributions was assessed using the Mauchly test. A Greenhouse–Geisser correction was used when sphericity was violated. A two-way ANOVA (3 conditions   ×   3 repetitions) was conducted to examine the main and interaction effects of condition and repetition. When significant effects were observed, Bonferroni-corrected *post hoc* tests were performed.

To examine the relationship between RPE and velocity loss, a linear mixed-effects model was fitted with velocity loss as the dependent variable and RPE as a fixed effect, and with participant included as a random effect with both random intercepts and random slopes (unstructured covariance). This specification accounts for the repeated-measures structure of the data, is robust to departures from normality, and allows the RPE–velocity-loss relationship to differ between individuals. Degrees of freedom were estimated using the Satterthwaite approximation and model assumptions were checked by inspection of the residual and random-effect distributions. From the fitted fixed-effect relationship, the RPE value corresponding to a velocity loss of 10% was derived. Marginal and conditional R^2^, and the intraclass correlation coefficient, are reported as measures of explained variance. We note that the CR-10 scale is ordinal rather than continuous, and the model estimates are therefore interpreted with corresponding caution. Statistical Analysis was conducted using SPSS version 31.0 (IBM Corp., Armonk, NY, USA). The level of statistical significance was set at *p* < 0.05.

## Results

**Figure 1:**
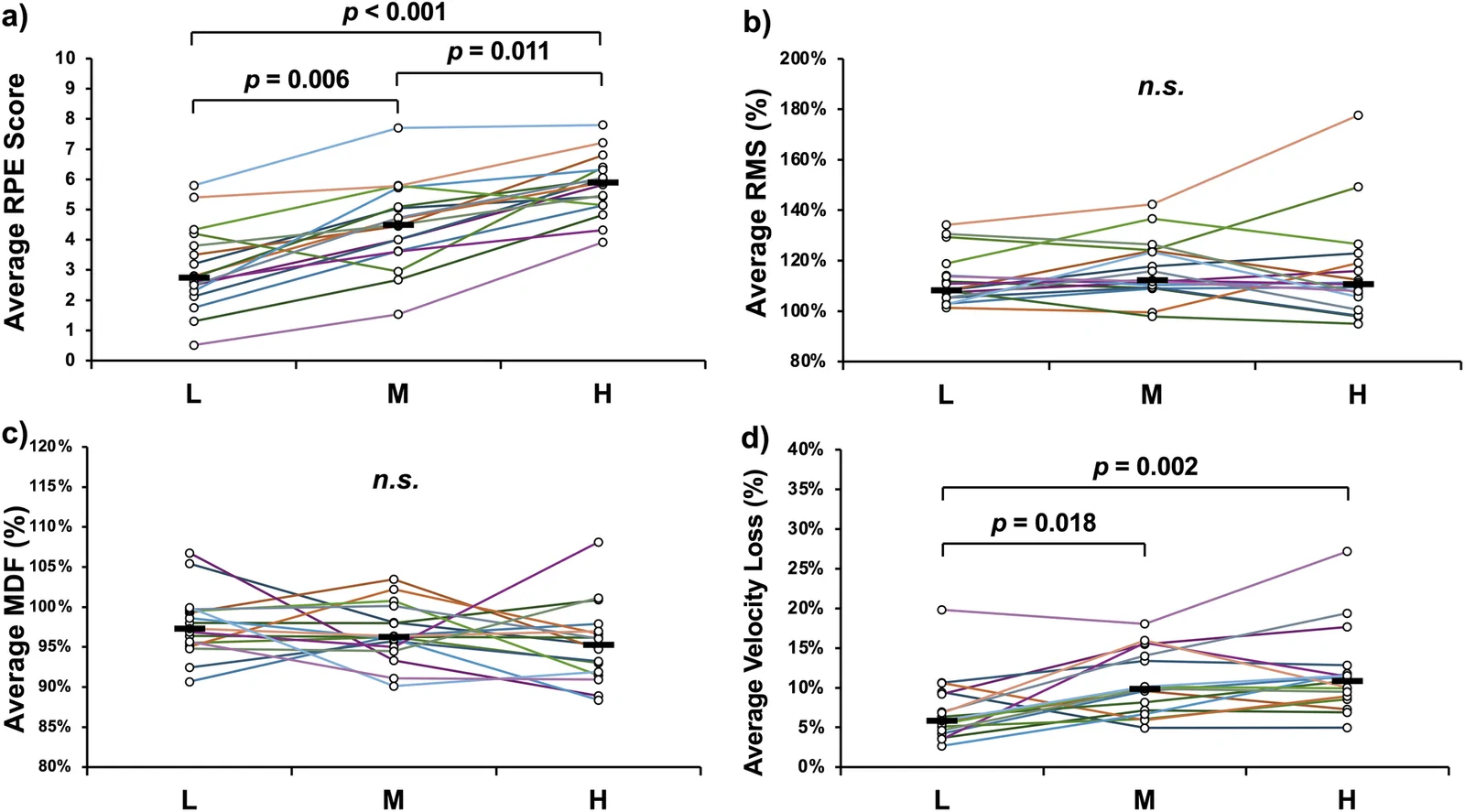
Average ratings of perceived exertion (RPE) **(a)**, average root mean square (RMS) **(b)**, average median frequency (MDF) **(c)**, and average velocity loss of back squat tasks. The *p* value represents the differences and significant level between experimental conditions.

Significant differences in average RPE were observed across the experimental conditions (*p* < 0.001, *F* = 20.073, partial *η^2^* = 0.455). *Post hoc* comparisons revealed significant differences between the L (95%CI [2.399, 3.645]) and M (95%CI [3.843, 5.088]) (*p* = 0.006), M and H (95%CI [5.174, 6.420]) (*p* = 0.011), and L and H (*p* < 0.001) conditions (Figure 1a). Significant differences in average velocity loss were observed across the experimental conditions (*χ^2^*(2) = 13.176, *p* < 0.001, Kendall's *W* = 0.388). *Post hoc* comparisons indicated significant differences in average velocity loss between the L and M (*p* = 0.018) and L and H (*p* = 0.002) conditions (Figure 1d). No significant differences in average RMS (*χ^2^*(2) = 0.824, *p* = 0.662, Kendall's *W* = 0.024) or MDF (*p* = 0.270, *F* = 1.344, partial *η^2^* = 0.053, 95%CI for L [0.957, 0.998]; 95%CI for M [0.946, 0.987]; 95%CI for H [0.933, 0.975]) were observed across the three experimental conditions (Figures 1b,c).

**Figure 2:**
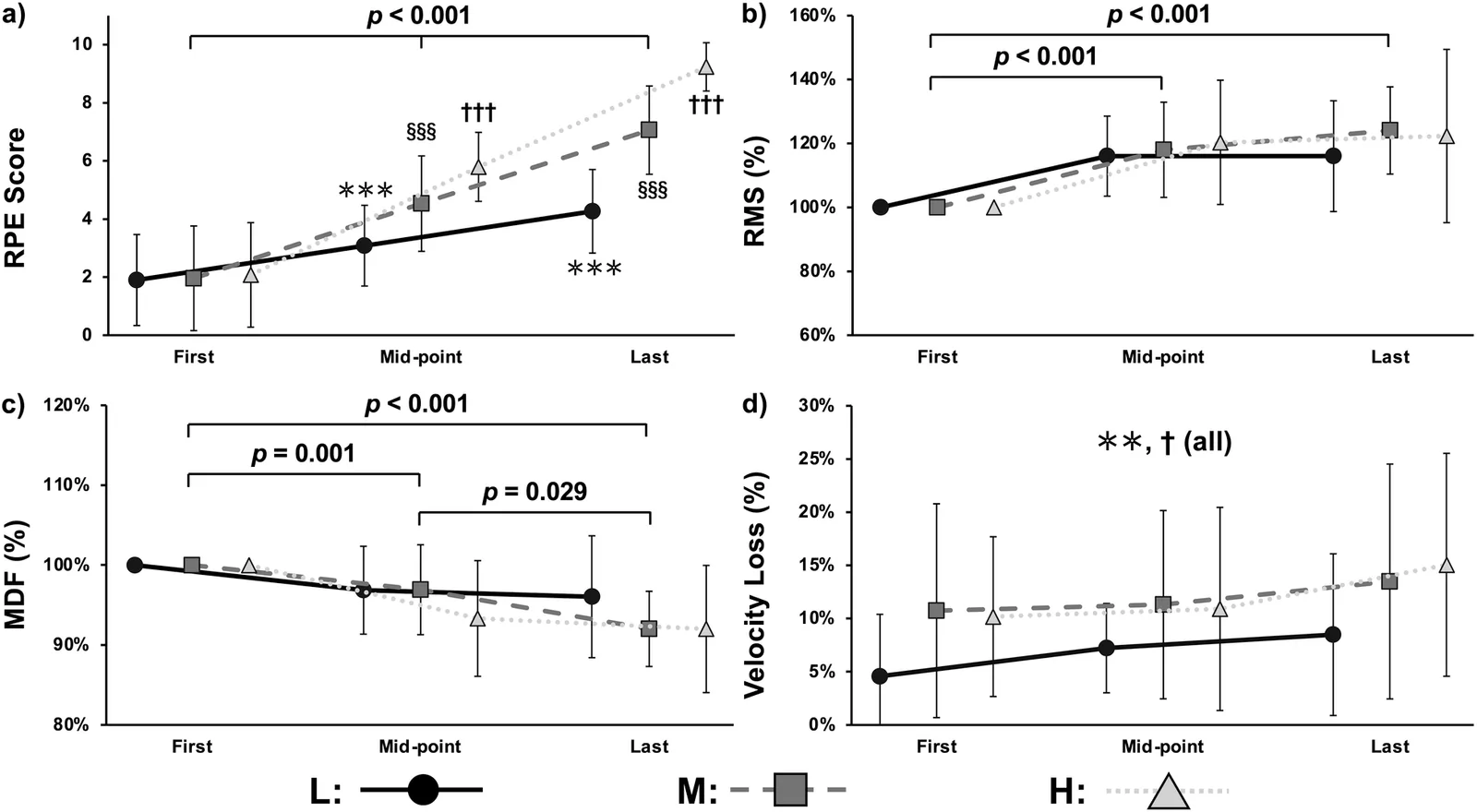
Rating of perceived exertion (RPE) **(a)**, root mean square (RMS) **(b)**, median frequency (MDF) **(c)**, and velocity loss during low (L, circle with solid lines), medium (M, square with dashed lines), and high (H, triangle with dotted lines) volume condition of exercises. * represents a significant difference as L compared with M condition, ** *p* < 0.01, *** *p* < 0.001; § represents a significant difference as M compared with H condition, §§§ *p* < 0.001; † represents a significant difference as L compared with H condition, † *p* < 0.05, ††† *p* < 0.001; The *p* value indicates difference between repetitions and its significant levels.

Intra-set data were analyzed using a two-way ANOVA, with the first, midpoint, and final repetitions extracted for comparison. A significant main effect of repetition was also found for RPE (*p* < 0.001, *F* = 151.600, partial *η^2^* = 0.905), RMS (*p* < 0.001, *F* = 23.595, partial *η^2^* = 0.600, 95%CI for L [1.000, 1.000]; 95%CI for M [1.112, 1.251]; 95%CI for H [1.122, 1.294]), and MDF (*p* < 0.001, *F* = 31.042, partial *η^2^* = 0.660, (95%CI for L [1.000, 1.000]; 95%CI for M [0.936, 0.978]; 95%CI for H [0.917, 0.950]), indicating consistent changes with increasing repetition number. No significant increase in velocity loss was observed during the lifting progression. A significant condition   ×   repetition interaction effect was observed for RPE (*p* < 0.001, *F* = 60.639, partial *η^2^* = 0.791). Between-condition comparisons revealed significant differences in RPE at the midpoint (*p* < 0.001, *F* = 52.173, partial *η^2^* = 0.765, 95%CI for L [2.368, 3.797]; 95%CI for M [3.685, 5.373]; 95%CI for H [5.184, 6.404]) and the final repetition (*p* < 0.001, *F* = 82.219, partial *η^2^* = 0.837, 95%CI for L [3.526, 5.004]; 95%CI for M [6.278, 7.840]; 95%CI for H [8.808, 9.663]). For velocity loss, a significant effect for conditions was observed (*p* < 0.001, *F* = 8.588, partial *η^2^* = 0.349). Between-condition comparisons revealed significant differences in velocity loss between the L (95%CI [0.048, 0.087]) and M (95%CI [0.084, 0.153]) (*p* = 0.007), and L and H (95%CI for H [0.091, 0.150]) (*p* = 0.012). No significant condition   ×   repetition interaction effect was observed. Additionally, no significant between-condition differences were found for intra-set RMS (*p* = 0.327, *F* = 1.158, partial *η^2^* = 0.067, 95%CI for L [1.064, 1.150]; 95%CI for M [1.093, 1.188]; 95%CI for H [1.062, 1.222]) or MDF (*p* = 0.189, *F* = 1.825, partial *η^2^* = 0.102, 95%CI for L [0.957, 0.995]; 95%CI for M [0.947, 0.979]; 95%CI for H [0.928, 0.975]) (Figure 2).

**Figure 3:**
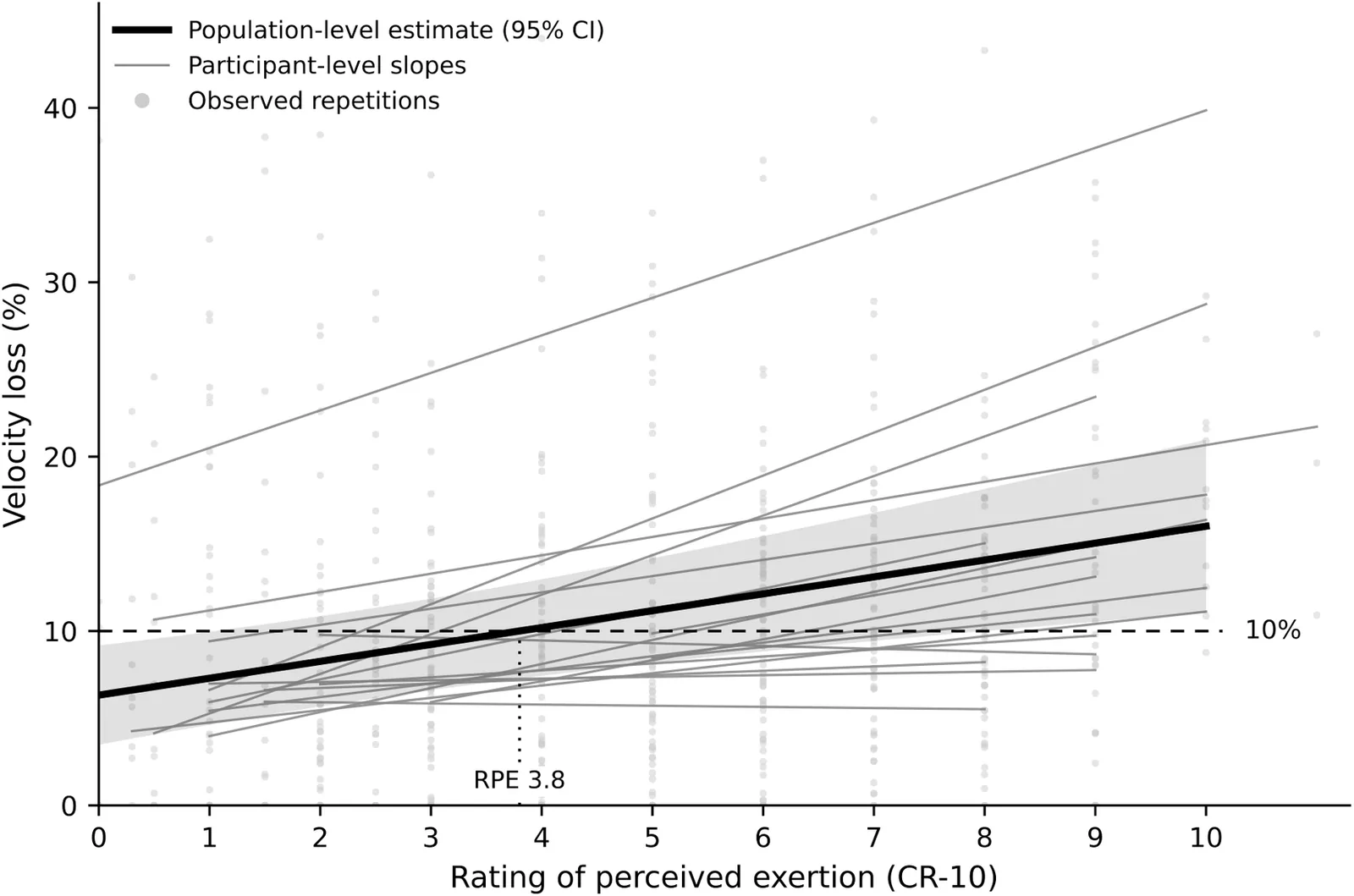
Modelled relationship between rating of perceived exertion (RPE) and velocity loss. Thin grey lines represent participant-level fitted slopes derived from the linear mixed-effects model; the thick black line represents the population-level (fixed-effect) estimate with its 95% confidence band. Grey points show the observed repetitions. The horizontal dashed line indicates a velocity loss of 10% and the vertical dotted line the corresponding RPE value of 3.8.

RPE was a significant predictor of velocity loss (*β* = 0.0097, SE = 0.0025, 95% CI 0.0043 to 0.0151, *t*(14.9) = 3.80, *p* = 0.002; Type III F (1, 14.9) = 14.46, *p* = 0.002), indicating that each one-unit increase in RPE corresponded to an increase in velocity loss of approximately 0.97 percentage points. The model intercept was 0.063 (SE = 0.013, 95% CI 0.035 to 0.091, *t*(18.2) = 4.74, *p* < 0.001). Estimated marginal means derived from the model indicated predicted velocity losses of 0.112 (11.2%; 95% CI 0.082 to 0.142) at an RPE of 5, 0.121 (12.1%; 0.088 to 0.155) at an RPE of 6, 0.131 (13.1%; 0.094 to 0.168) at an RPE of 7, and 0.141 (14.1%; 0.100 to 0.182) at an RPE of 8. Inverting the fixed-effect relationship, a velocity loss of 10% corresponded to an RPE of 3.8. Both random effects were statistically significant. The variance of participant intercepts was 0.0021 (SE = 0.0009, Wald Z = 2.21, *p* = 0.027; SD = 0.046) and the variance of participant slopes was 0.00008 (SE = 0.00004, Wald Z = 2.03, *p* = 0.042; SD = 0.0089), whereas the intercept–slope covariance did not differ from zero (Wald Z = −0.55, *p* = 0.580). The residual variance was 0.0046 (SE = 0.0003, Wald Z = 15.89, *p* < 0.001). The standard deviation of the participant slopes (0.0089) was of a similar magnitude to the fixed-effect slope itself (0.0097), indicating that the strength of the RPE–velocity-loss relationship differed substantially between participants. The marginal R^2^ was 0.073 and the conditional R^2^ was 0.483, with an intraclass correlation coefficient of 0.442, indicating that a greater proportion of the variance in velocity loss was attributable to differences between participants than to RPE itself. Full model parameters are presented in Table 1, and the modelled relationship, including participant-level slopes, is shown in Figure 3.

**Table 1:** Linear mixed-effects model of velocity loss predicted by rating of perceived exertion.

Fixed effects						
Parameter	Estimate	SE	*df*	*t*	*p*	95%CI
Intercept	0.063	0.013	18.2	4.74	<0.001	0.035, 0.091
RPE	0.0097	0.0025	14.9	3.80	0.002	0.0043, 0.0151

Random effects and residual						
Parameter	Estimate	SE	Wald *Z*	*p*	95%CI	SD
Intercept variance	0.0021	0.0009	2.21	0.027	0.0009, 0.0051	0.046
Slope (RPE) variance	0.00008	0.00004	2.03	0.042	0.00003, 0.00021	0.0089
Intercept–slope covariance	−0.0001	0.0002	−0.55	0.580	−0.0004, 0.0002	—
Residual	0.0046	0.0003	15.89	<0.001	0.0041, 0.0052	0.068

Model fit						
Index				Value		
−2 restricted log-likelihood				−1278.83		
AIC				−1270.83		
BIC				−1253.69		
Marginal *R^2^*				0.073		
Conditional *R^2^*				0.483		
ICC				0.442		

Velocity loss is expressed as a proportion. The model included participant as a random effect with unstructured covariance for random intercepts and slopes. Degrees of freedom were estimated using the Satterthwaite approximation.

CI, confidence interval; ICC, intraclass correlation coefficient; RPE, rating of perceived exertion.

## Discussion

The purpose of this study was to (1) examine the criterion-related validity of RPE as an indicator of velocity loss by modelling the within-participant RPE–velocity-loss relationship, and (2) characterize neuromuscular activation responses during power BS under different repetition settings. The key findings of the study are as follows: (1) average RPE and average velocity loss both differed across the experimental conditions, and RPE was a significant predictor of velocity loss within individuals; specifically, a velocity loss of 10% corresponded to an RPE of 3.8. (2) No significant between-condition differences were observed in either intra-set or average values of RMS and MDF. This absence of differences may partly reflect the limited muscle sampling rather than a genuine physiological characteristic of the lower-limb muscles during power BS. (3) Velocity loss was significantly lower in the L condition, indicating that, during power training, prescribing repetition numbers below 30% (Here, ‘30%’ refers to 30% of the repetitions completed to volitional failure, rather than to a percentage of velocity loss) may minimize fatigue-induced reductions in velocity and power output. We acknowledge that, at the intensity examined here (65% 1RM), current guidelines already recommend low repetition volumes for power-oriented training (2), and that these recommendations broadly correspond to our low-repetition condition. The repetition threshold itself is therefore not presented as the principal contribution of this study. Rather, the contribution lies in quantifying the relationship between a perceptual marker and velocity loss, and in expressing that relationship as RPE values corresponding to defined velocity-loss thresholds: an RPE of 3.8 corresponded to a 10% velocity loss.

Perceived exertion during resistance training is influenced by a number of central and peripheral physiological responses (28, 39). For example, previous studies have reported a close relationship between RPE and central motor output (39–41). With respect to peripheral responses, RPE has been shown to correlate significantly with metabolic and endocrine markers, such as blood lactate and testosterone concentrations (4, 42). In this study, decreases in MDF were attributed to reductions in muscle fiber conduction velocity, which are influenced by disruptions in homeostasis, such as pH reduction and the accumulation of metabolic byproducts (33, 43–45). Furthermore, these peripheral changes increase afferent feedback from the working muscles, thereby elevating RPE (46, 47). It should be noted that these significant changes in RMS and MDF refer to repetition (within-set) effects: as the repetition number increased within each set, RMS increased significantly and MDF decreased significantly, whereas neither variable differed significantly between the three lifting conditions. The within-set increase in RMS indicates a marked augmentation of central nervous system drive (39), and in field-based studies using sEMG, neuromuscular fatigue is commonly identified as concurrent significant changes in RMS and MDF (10, 48). These concomitant changes in MDF and RMS suggest that neuromuscular fatigue was induced during the lifting process and that RPE was consequently elevated. However, no significant between-condition differences in neuromuscular fatigue were detected based on RMS or MDF. This finding is consistent with previous related work (49, 50). It has been reported that fatigue patterns differ between upper- and lower-body muscles due to their distinct physiological characteristics. We failed to directly assess metabolic-related responses and histological characteristics, such as blood lactate and muscle fiber composition in present study, and this remains a possible explanation for the absence of between-condition differences in RMS/MDF. Given the association between spectral measures and motor-unit conduction velocity (11) and the absence of between-condition differences in MDF, present study only provides a rudimental insight on neuromuscular fatigue in power-aimed BS. Because the three conditions were performed in a randomized order, the present data do not describe a progressive change across successive conditions; rather, RPE and velocity loss differed among the L, M, and H conditions as reported above.

When focusing on average velocity loss across conditions and on selected repetitions during the lifting process, trends different from those of neuromuscular fatigue were observed. For strength and conditioning coaches aiming to improve explosive performance, such as velocity and power, it is crucial to instruct athletes to lift with maximal intended velocity and to avoid excessive velocity loss (7, 26). During training, neuromuscular fatigue is inevitable, and velocity and power output consequently decrease (7, 12, 51). However, velocity assessment devices (e.g., GymAware) are often unavailable to individual users due to barriers such as cost, precision requirements, and limited interoperability (12, 52). The present study provides a preliminary suggestion for practitioners, such as coaches and trainers who are unable to use velocity assessment devices during power training. For practitioners without access to velocity-monitoring devices, the present data indicate that a reported RPE of approximately 3.8 corresponds, on average, to a 10% velocity loss during power BS at a moderate intensity. This provides a device-free reference point that can be incorporated into existing autoregulation practice. Because the relationship varied substantially between participants, however, this value should be calibrated individually rather than applied uniformly, and further evidence is required before it can be recommended for long-term programming.

An interesting finding of the present study is that RPE scores exhibited between-condition differences similar to those observed for velocity loss across all comparisons. Further, RPE was significantly associated with velocity loss within individuals during power BS exercise, and RPE values corresponding to defined velocity-loss thresholds (an RPE of 3.8 for a 10% velocity loss). This result indicates that higher RPE was associated with greater velocity loss across conditions. Although RPE cannot directly replace linear position transducers, it may provide useful information regarding fatigue-related performance decrements during power back squat. It has been reported that RPE and velocity change in a similar manner; however, submaximal set configurations can substantially influence their relationship, which may, in turn, affect neuromuscular fatigue responses (26). These findings are consistent with previous studies focusing on explosive exercises, including the BS and bench press (21, 41). Although the maximal number of repetitions has been reported to be influenced by factors such as sex, training experience, and current training status (53), each participant served as their own reference in the present design. This supports our interpretation that the RPE–velocity-loss relationship can be characterized within individuals, although its strength differed considerably between them. Accordingly, we conclude that RPE and velocity loss are influenced by set length, whereas neuromuscular fatigue is not substantially affected by such modifications in set configuration.

This finding also provides a new practical perspective for velocity-based training. In practice, the approach would involve three steps: the athlete first performs one set to volitional failure at the prescribed load to anchor the upper limit of perceived exertion; RPE is then reported at predetermined repetitions during subsequent sets; and the set is terminated when the reported value reaches the RPE associated with the chosen velocity-loss threshold. Beyond the single anchoring set, this adds only a brief verbal report per set, which is comparable in burden to RPE-based autoregulation as it is already practiced. We emphasize, however, that because of the between-participant variability observed here, the reference values should be calibrated individually rather than applied uniformly. These values should, however, be interpreted with appropriate caution. RPE accounted for a modest proportion of the variance in velocity loss at the population level (marginal R^2^ = 0.073), and most of the variance was attributable to differences between participants (ICC = 0.442). The cut-offs reported here are therefore population-level reference values rather than precise individual predictions, and they are not a substitute for device-based velocity monitoring. The RPE–velocity-loss relationship varied considerably between participants in both intercept and slope, and the variance of the participant slopes was statistically significant. RPE should therefore not be regarded as uniformly tracking velocity loss at the individual level, as illustrated in Figure 3. Their practical value lies in providing an approximate, individually calibrated reference for practitioners who do not have access to velocity-monitoring equipment. Individual calibration — for example, establishing a personal reference during an initial set at the prescribed load — remains necessary before the reported values are applied in practice.

This study has several limitations on experimental design. First, only two thigh muscles (the vastus lateralis and vastus medialis) were analyzed for neuromuscular fatigue quantification. The distinct physiological characteristics of lower-limb muscle (e.g., fiber-type composition and lactate kinetics) may contribute to the absence of between-condition differences in neuromuscular fatigue (49, 50). In addition, marked muscle fatigue may have occurred in muscles not examined in the present study (e.g., the lower back). Second, only high-speed video from the sagittal plane was recorded, which may have limited measurement precision compared with three-dimensional motion capture systems (12, 13). Further, 2D tracking captures less information than 3D tracking, which may introduce additional measurement error (Pérez-Castilla et al., 2019; Külkamp et al., 2023) Third, the anchoring trial was performed only once, so we cannot rule out between-session variation in the maximal repetition number, which may affect the results (54). Fourth, although a previously recommended rest interval was set between conditions, all three conditions were implemented in a single session, and residual fatigue between conditions may have affected the results. Fifth, present study comprised only young, resistance-trained males, and the findings should not be generalized to females, older adults, novice lifters or rehabilitation populations. Future studies should focus on assessing a greater number of muscles, applying gold-standard methods for velocity measurement, and examining the long-term effects of the repetition threshold observed in the present study. Several limitations on statistical analysis should also be noted in relation to the modelling. First, the CR-10 scale is ordinal rather than continuous, and the model estimates should be interpreted with this in mind. Second, RPE explained only a modest proportion of the variance in velocity loss (marginal R^2^ = 0.073), and the relationship varied substantially between participants; the reported cut-off values are therefore population-level estimates requiring individual calibration. Third, the present analysis quantifies the association between RPE and velocity loss but does not establish agreement between them; formal agreement analysis is required before RPE could be regarded as interchangeable with device-based measurement. Fourth, the model intercept (0.063) reflects the fact that the repetition-wise velocity-loss index does not begin at zero, because the fastest repetition was not necessarily the first.

## Conclusion

The present study indicated that both RPE and velocity loss differed across the repetition settings examined. Further, RPE was significantly associated with velocity loss within individuals during power BS exercise with a moderate load (i.e., 65% 1RM), and this relationship could be expressed as an RPE of 3.8 corresponding to a 10% velocity loss. These values offer a device-free reference that can be incorporated into established RPE-based autoregulation of repetition volume at a fixed load. Because RPE explained only a modest proportion of the variance in velocity loss and the relationship differed substantially between participants, the values reported here should be regarded as population-level references requiring individual calibration, and as preliminary until prospectively validated. Neuromuscular activation indices (RMS and MDF) changed within a set but did not differ between repetition conditions; given the limited muscle sampling, these indices should be interpreted with caution.

## Data Availability

The raw data supporting the conclusions of this article will be made available by the authors, without undue reservation.
